# Distinguishing features of *Lycium* L. species (family Solanaceae) distributed in Egypt based on their anatomical, metabolic, molecular, and ecological characteristics

**DOI:** 10.3389/fpls.2023.1162695

**Published:** 2023-05-12

**Authors:** Osama G. Ragab, Diaa Mamdouh, Ramadan Bedair, Iryna Smetanska, Nazim S. Gruda, Sawsan K. M. Yousif, Rihab M. Omer, Ashwaq T. Althobaiti, Hany S. Abd El-Raouf, Ahmed M. El-Taher, Ashraf S. El-Sayed, Marwa M. Eldemerdash

**Affiliations:** ^1^ Department of Botany and Microbiology, Faculty of Science, Al-Azhar University, Cairo, Egypt; ^2^ Department of Plant Food Processing, Agricultural Faculty, University of Applied Sciences Weihenstephan-Triesdorf, Weidenbach, Germany; ^3^ Division of Horticultural Sciences, Institute of Crop Science and Resource Conservation, University of Bonn, Bonn, Germany; ^4^ Department of Chemistry, College of Arts and Science in Baljurashi, Al-Baha University, Al Bahah, Saudi Arabia; ^5^ Department of Biology, College of Science, Taif University, Taif, Saudi Arabia; ^6^ Department of Agricultural Botany, Faculty of Agriculture, Al-Azhar University, Cairo, Egypt; ^7^ Department of Biology, University College, Taif University, Taif, Saudi Arabia; ^8^ Botany and Microbiology Department, Faculty of Science, Zagazig University, Zagazig, Egypt

**Keywords:** *Lycium*, taxonomic, ITS sequence, SCoT analysis, GC-MS, ecology

## Abstract

Among the 70–80 species of the genus *Lycium* (family Solanaceae) disjunctly distributed around the world, only three are frequently distributed in different locations in Egypt. Due to the morphological similarities between these three species, there is a need for alternative tools to distinguish them. Thus, the objective of this study was to revise the taxonomic features of *Lycium europaeum* L., *Lycium shawii* Roem. & Schult., and *Lycium schweinfurthii* var. *aschersonii* (Dammer) Feinbrun in consideration of their anatomical, metabolic, molecular, and ecological characteristics. In addition to analysis of their anatomical and ecological features, DNA barcoding was performed for molecular characterization through internal transcribed spacer (ITS) sequencing and start codon targeted (SCoT) markers. Furthermore, metabolic profiling of the studied species was conducted based on gas chromatography–mass spectrometry (GC-MS). The observed anatomical features of the adaxial and abaxial epidermal layers, type of mesophyll, crystals, number of palisade and spongy layers, and the vascular system showed variations between the studied species. Beyond this, the anatomy of the leaves showed an isobilateral structure in the studied species, without distinct differences. Species were molecularly identified in terms of ITS sequences and SCoT markers. The ITS sequences were deposited in GenBank with accession numbers ON149839.1, OP597546.1, and ON521125.1 for *L. europaeum* L., *L. shawii*, and *L. schweinfurthii* var. *aschersonii*, respectively. The sequences showed variations in GC content between the studied species; this was 63.6% in *L. europaeum*, 61.53% in *L. shawii*, and 63.55% in *L. schweinfurthii* var. *aschersonii*. A total of 62 amplified fragments, including 44 polymorphic fragments with a ratio of 70.97%, were obtained in the SCoT analysis, as well as unique amplicons in *L. europaeum L.*, *shawii*, and *L. schweinfurthii* var. *aschersonii* of 5, 11, and 4 fragments, respectively. Through GC-MS profiling, 38 compounds were identified with clear fluctuations in the extracts of each species. Of these, 23 were distinguishing chemicals that could help in chemical identification of the extracts of the studied species. The present study succeeds in identifying alternative clear and diverse characteristics that can be used to distinguish between *L. europaeum*, *L. shawii*, and *L. schweinfurthii* var. *aschersonii*.

## Introduction

The genus *Lycium* L. belongs to the family Solanaceae, which comprises approximately 70–80 species. These are distributed around the world in temperate to subtropical regions, mainly in North America, South America, southern Africa, Eurasia, and Australia (Hunziker, 1979; Bernardello, 1986; Fukuda et al., 2001; Levin and Miller, 2005). In Egypt, three species of *Lycium* have been studied, namely, *Lycium europaeum* L., *Lycium shawii* Roem. & Schult., and *Lycium schweinfurthii* var. *aschersonii* (Dammer) (Boulos, 2002). The anatomical features of the internal phloem of the Solanaceae family have been reported for the European *Lycium chinense* Mill., including its histogenesis and development (Fukuda, 1967). In addition, the distinctive taxonomic and anatomical features of the Solanaceae family have been frequently described: by Metcalfe and Chalk (1979), by Hawkes et al. (1979), by Watson and Dallwıtz (1992), and by Norverto (2000). Extensive phylogenetic and biogeographical studies of *Lycium* have been carried out by Fukuda et al. (2001). Selvi et al. (2009) described the anatomical structures of the stem and leaf of the endemic *Lycium anatolicum* distributed in Turkey. Morphological and anatomical studies of *L. chinense* and *Lycium barbarum* L., with an obvious calcium oxalate in the form of brown crystalline sand, have been reported (Alikarieva, 2022). The family Solanaceae is rich in compounds with distinct floristic, phytochemical, economic, and ethnobotanical significance (Hawkes et al., 2000).

*Lycium* species have been recognized as containing a repertoire of various compounds with multiple pharmaceutical and biological activities (Yao et al., 2011; Qian et al., 2017). In Egypt, less attention has been paid to morphological, metabolic, and molecular analyses of *Lycium* species, despite the diverse pharmaceutical applications of the metabolites derived from these plants. Taxonomic analyses based on the traditional anatomical and morphological features of the plants have been revised and authenticated for various plant species, including *Cestrum* (Solanaceae), seven taxa of the subfamily Caesalpinioideae (El-Demerdash et al., 2021; Eldemerdash et al., 2022), and *Solanum* (Solanaceae) (El-Shaboury et al., 2017). Recently, several molecular markers, such as amplified microsatellite polymorphism PCR, inter-simple sequence repeat (ISSR) markers, and random amplified polymorphic (RAPD) markers, as well as start codon targeted (SCoT) polymorphisms, have been used in molecular confirmation of traditional taxonomic features (Deng et al., 2017; Abd El-Ghani et al., 2021). The major advantages of the use of molecular markers to confirm traditional taxonomic features include their simplicity and their highly polymorphic nature, requiring very low DNA concentration (Xiong et al., 2009).

Based on the literature, molecular identification, DNA barcoding, and metabolic profiling of *Lycium* sp. have received less attention. Molecular identification and DNA barcoding analyses have become crucial taxonomic approaches for the verification and revision of the traditional taxonomic features of plants due to their precision, reproducibility, efficiency, and technical feasibility (Cahyaningsih et al., 2022; Kress and Erickson, 2007; Kress et al., 2014). The accuracy and reproducibility of molecular tools for plant identification can mainly to attributed to their independence of the morphological diversity of species and climatic and environmental conditions (Newmaster et al., 2013; Techen et al., 2014). The internal transcribed spacer (ITS) region of eukaryotic nuclear ribosomal DNA (rDNA), which contains two internal transcribed spacers (ITS1 and ITS2) and the 5.8S subunit, plays a significant role in systematic molecular investigations of flowering plants (Baldwin et al., 1995). The sequences of the rDNA regions of the plant genome, in terms of large and small subunits of rRNA, have been used as universal molecular markers and barcodes for plant identification (Almerekova et al., 2017). Among these rDNA sequences, ITS1 and ITS2 have been used as universal barcodes for plant identification (Wu et al., 2013; Kim et al., 2015).

In addition to the use of molecular tools, numerous recent studies have emphasized the combination of taxonomic studies and molecular barcoding with gene expression analysis, especially metabolic profiling patterns. Among the different metabolic approaches, volatile metabolites have been recognized as one of the characteristic taxonomic features for plant identification. Gas chromatography–mass spectrometry (GC-MS) has become recognized as a crucial approach for determining the various volatile compounds present in plants, particularly non-polar components, volatile essential oils, fatty acids, esters, and long-chain branch hydrocarbons (Chandrasekaran et al., 2010; Asraoui et al., 2021). Phytochemical screening with GC-MS metabolic profiling of plants has recently been used as one of a set of robust taxonomic tools, as has been reported for species of *Cestrum* (Solanaceae), seven taxa of the subfamily Caesalpinioideae (El-Demerdash et al., 2021; Eldemerdash et al., 2022), and 11 species of *Solanum* (Solanaceae) (El-Shaboury et al., 2017). GC-MS has been extensively used in the identification of bioactive compounds from plants with pharmaceutical and technological bioactivity (Wang et al., 2018; Selvaraj et al., 2020). Although metabolic profiling of plants has been frequently implemented as a tool for the confirmation of traditional taxonomic features, the metabolic profiling of *Lycium* species and their correlation with taxonomic features has received less attention in Egypt.

Unfortunately, only a few taxonomic and anatomical studies of *Lycium* species have been conducted in Egypt. Moreover, *L. europaeum* and *L. schweinfurthii* have been reported to be poorly studied species of the genus *Lycium* worldwide, although they are distributed in several countries. In the last 5–8 years of research, they have attracted attention for their chemical constituents, especially their probable production of valuable secondary products (Miguel, 2022). The importance of *L. schweinfurthii* as a source of phenolics and flavonoid compounds with high antioxidant activity has been reported (Mamdouh et al., 2021). Furthermore, 30 compounds have been identified in *L. europaeum*, including compounds from several important groups, namely polyphenols, polysaccharides, carotenoids, sterols, and alkaloids (Wannes and Tounsi, 2021). Beyond this, interest in studying the importance of *L. shawii* arose earlier than interest in the other two species. The antioxidant and microbial activities of the fruits of the former have been reported (Dahech et al., 2013), and their antidiabetic activity and toxic potential have been evaluated in mice (Sher and Alyemeni, 2011). New glucosides and secondary metabolites have been identified and isolated from *L. shawii* (Rehman et al., 2017; Ali et al., 2020) and *L. schweinfurthii* (Elbermawi et al., 2019; Elbermawi et al., 2022). Through the phytochemical screening of *L. europaeum*, the presence of phenolics, flavonoids, alkaloids, sesquiterpenes, and tannins showing antioxidant, anticancer, and anti-inflammatory activities has been reported (Bendjedou et al., 2019; Mejri et al., 2022). Moreover, the anti-nociceptive, hepatoprotective, and nephroprotective effects of *L. europaeum* have been observed in mice (Rjeibi et al., 2017).

Thus, the objective of this study was to revise the traditional taxonomic and ecological features of the different *Lycium* species in Egypt in light of recent molecular tools for DNA barcoding and metabolic profiling.

## Materials and methods

### Collection of plant samples

**Figure 1:**
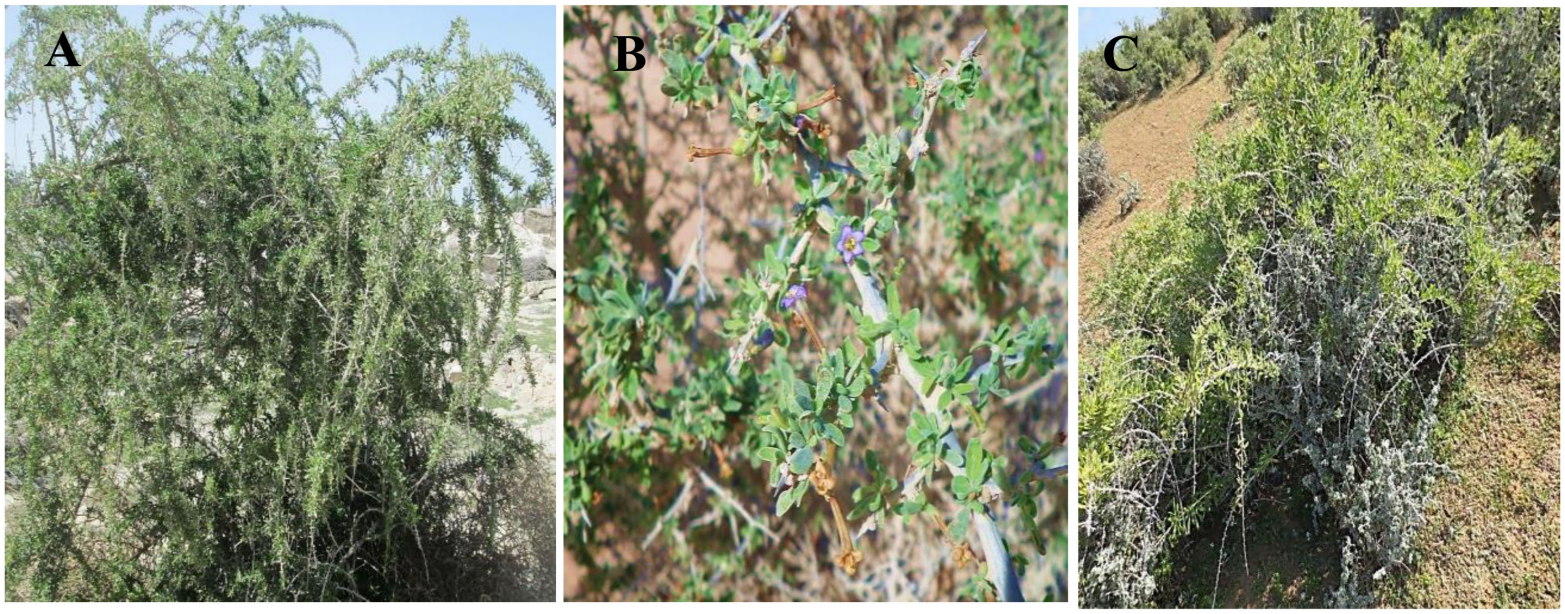
The studied *Lycium* species at the collection sites. **(A)** *Lycium europaeum*. **(B)** *Lycium shawii*. **(C)** *Lycium schweinfurthii* var. *aschersonii*.

**Figure 2:**
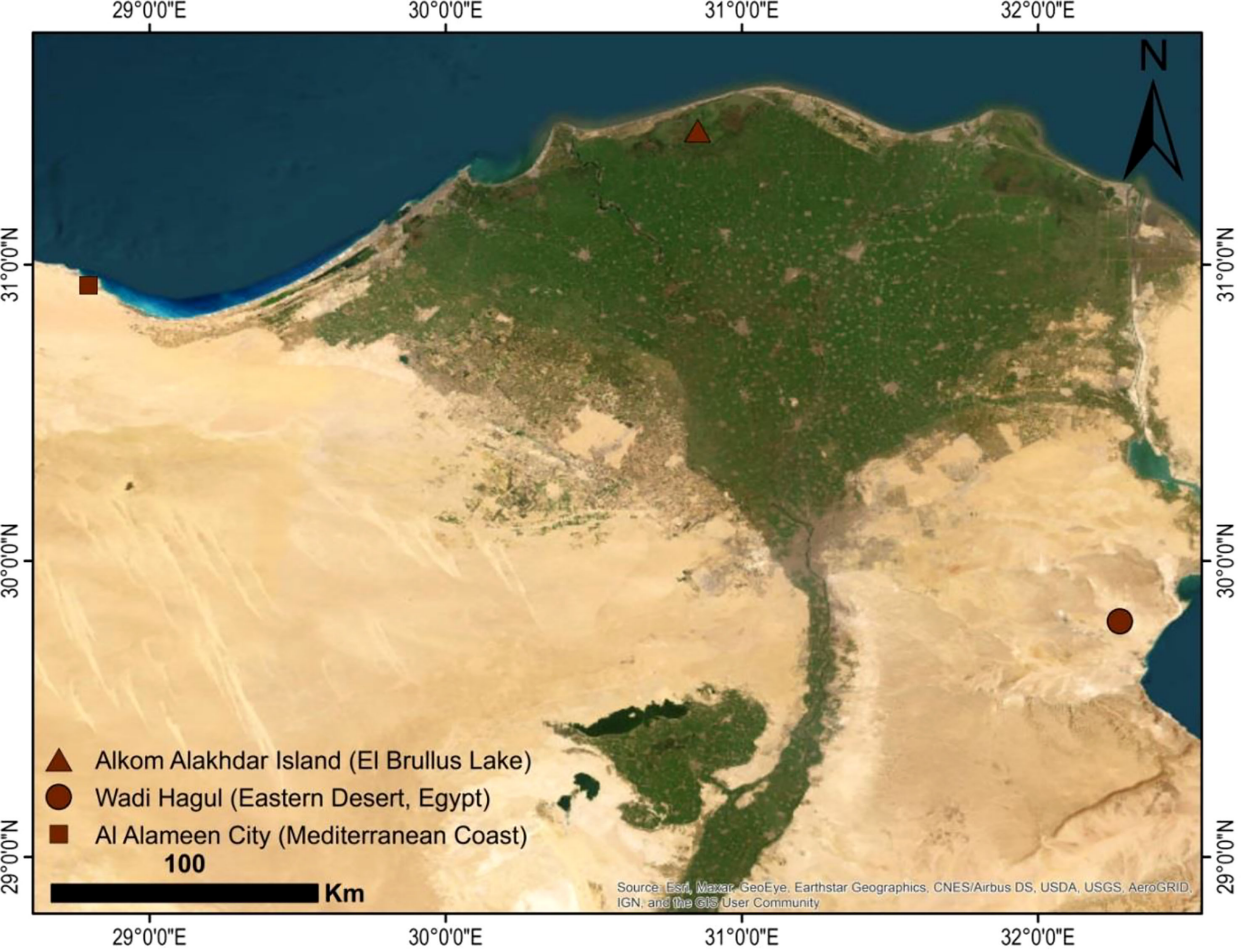
Map of the area studied according to Arc-GIS 10.5.

Three wild species of *Lycium*, namely, *L. europaeum*, *L.*, *shawii* Roem. & Schult., and *L. schweinfurthii* var. *aschersonii* (Dammer) Feinbrun (**Figure 1**), were studied. A total of 15 stands were selected for the study of the floristic composition of the *Lycium* communities during the spring season of 2019 and 2020. In each stand, four quadrates were chosen from different localities in Egypt (quadrate area = 10 × 10 = 100 m^2^) (**Figure 2**). The life span each of the studied species stands was established based on Täckholm (1974) and Boulos (1999; 2000; 2005; 2009). The life form and floristic category were recognized by Raunkiaer (1934) and Tutin et al. (1964). Plants were identified based on traditional morphological features according to the keys of Täckholm (1974) and Boulos (2002). Specimens of the studied species were prepared and kept in the herbarium of the Department of Botany and Microbiology, Faculty of Science, Al-Azhar University, Cairo, Egypt (**Table 1**).

**Table 1:** Date and site of collection of *Lycium* species.

Species	Collection date	Collection site
*Lycium europaeum* L.	March 2019	Alexandria—Matruh coastal road, 237 km
*Lycium shawii* Roem. & Schult.		Wadi Hagul
*Lycium schweinfurthii* var. *aschersonii* (Dammer) Feinbrun		Alkom Alakhdar Island, El Brullus

### Anatomy of the plants

The freshly collected leaves of the specimens were washed and fixed in formalin, glacial acetic acid, and 70% ethyl alcohol (5:5:90, *v*/*v*) following Nassar and El-Sahhar (1998). The leaves were then sectioned with a microtome at 12–15 µm, embedded in paraffin wax, and stained with safranin solution (Dilcher, 1974). The sections were examined and photographed using a light microscope (Optika, with premiere MA88-900 digital camera). The anatomical traits of the plant sections were determined according to Metcalfe and Chalk (1950). Data on the anatomical analyses of the leaves were scored and coded to build a numerical data matrix. Statistical analysis was conducted using PRIMER software (version 6.0) to compare the investigated taxa.

### Molecular fingerprinting analysis

The studied plants were molecularly identified based on their rDNA sequences flanking the ITS1, 5.8S, and ITS2 regions. DNA extraction of the studied *Lycium* species was performed with a CTAB lysis buffer according to Aboul-Maaty and Oraby (2019), with some modifications. Fresh weights of 100 mg of plant tissue from the three species were pulverized in liquid nitrogen. A volume of 500 µl of the CTAB buffer was added and the samples were vortexed for 1 min. After 10 min, the samples were centrifuged at 11,200 × *g*, chloroform was added to the supernatant, and this was shaken vigorously. The supernatant obtained after 10 min centrifugation (11,200 × *g*) was diluted with a double volume of absolute ethanol. The mixture was then incubated at −20°C for 30 min. After centrifugation, the pelleted DNA was dissolved in 50 μl of nuclease-free distilled water and stored at −20°C until use. Gel electrophoresis was performed to check the purity of the extracted DNA using 1% agarose gel against a 1-kb DNA ladder (cat. no. PG010-55DI).

The plant genomic DNA was used as a DNA template for the PCR reaction. The PCR reaction mixture contained 10 μl of 2× PCR master mixture (i-Taq™, cat. no. 25027), 2 μl of gDNA, and 10 pmol of primers (for ITS and SCoT, separately), completed to 20 μl with sterile distilled water (**Table 2**). PCR amplification was performed using a Thermal Cycler 006 under the same conditions as those used for the ITS and SCoT primers. The PCR reaction started with initial denaturation at 94°C for 2 min. Subsequently, 35 repeated cycles of denaturation at 94°C for 30 s, annealing at specific temperature for each primer (**Table 2**) for 30 s, and extension at 72°C for 1 min were performed. The reaction mixture was exposed to a final extension for 5 min at 72°C. The PCR amplicons of the ITS and SCoT regions for the different samples were analyzed with 1.5% agarose gel. The ITS regions of the plants were sequenced using an Applied Biosystem Sequencer (HiSQV Bases, version 6.0). The sequences were non-redundantly BLAST-searched on NCBI; the outputs were imported into MEGA X software and aligned using the ClustalW muscle algorithm. Phylogenetic relatedness was determined using the neighbor-joining (NJ) method (Saitou and Nei, 1987).

**Table 2:** List of the internal transcribed spacer (ITS) and start codon targeted (SCoT) primer sequences used in the study.

Primer name	Primer sequence (5′–3′)	Reference	Annealing temperature (°C)
ITS4	TAG AGGAAGGAGAAGTCGTAA CAA	Chen et al., 2010	51
ITS5	CCCGCCTGACCTGGGGTCGC		62
SCoT-01	CAACA**ATG**GCTACCACCA	Collard and Mackill, 2009	51
SCoT-02	CAACA**ATG**GCTACCACCC		52
SCoT-03	CA**ATG**GCTACCACTAGCG		51
SCoT-04	ACA**ATG**GCTACCACTAGG		49
SCoT-05	CAACA**ATG**GCTACCACGA		51
SCoT-06	CAACA**ATG**GCTACCACGC		52
SCoT-09	CAACA**ATG**GCTACCAGCA		49
SCoT-52	ACA**ATG**GCTACCACTGCA		52

The ITS sequences were adjusted using BioEdit, version 7.2.5 (Hall, 1999). The length and GC contents were estimated for each sequence using the EndMemo software (http://www.endmemo.com/bio/gc.php). The sequences were then analyzed manually. The pairwise sequence divergence between the studied taxa regarding the sequences of the ITS1, 5.8S, and ITS2 regions was calculated according to the maximum composite likelihood method (Tamura et al., 2007). The obtained sequences were verified by non-redundant search with BLAST tools. Gaps and missing data were excluded from the dataset. The values of the internal branches of the NJ tree were evaluated through the bootstrap method (1,000 replicates). Thereafter, the transition/transversion (*t*_i_/*t*_v_) ratio was calculated using the following formula: *R* = [A*G*k_1_ + T*C*k_2_]/[(A + G)*(T + C)], where A, G, C, and T represent the corresponding frequencies of the bases (Tamura et al., 2007).

Clear, reproducible, and intense bands of PCR fragments resulting from the use of primers were scored. The binary matrix of the amplified bands was recorded, and the data were then analyzed using the NTSYS-pc software, version 2.1 (Rholf, 2002). Jaccard’s similarity coefficients were determined and were used to generate a dendrogram using the unweighted pair group method with arithmetic average (UPGMA). The data were then used to generate a dendrogram, in accordance with Sneath and Sokal (1973), to represent the relationships between the studied species.

Additionally, the DNA of the studied species was exposed to PCR using SCoT primers. PCR was performed using 10 SCoT primers to analyze the genomic differences among the studied species. From these, only eight primers were selected based on the production of clear and reproducible fragments (**Table 2**). The banding pattern was recorded and analyzed, while the polymorphism percentage was calculated.

### Metabolic profiling of the studied plants by GC-MS analysis

Metabolic profiling of the different *Lycium* species was performed via GC-MS analysis. Volatile compounds were extracted from the plants using methanol according to the method by El-Demerdash et al. (2021), with slight modifications. Briefly, fresh healthy leaves were shade-dried for 2 weeks and pulverized into a dry powder using a household blender. In conical flasks, 10 g of the powdered leaves was extracted by cold maceration in 50 ml methanol (1:5, *w*/*v*) for 72 h at room temperature (Nandagopalan et al., 2015; Socrates and Mohan, 2019). The extracts were filtered with a muslin cloth, and the filtrates were collected and centrifuged for 10 min at 11,200 × *g*. The supernatant was collected and the solvent subsequently evaporated to a final volume of 5 ml. Thereafter, the vials were tightly sealed and kept at 4°C in darkness until use. The metabolic pattern of the volatile components was assessed using a GC-TSQ mass spectrometer (Thermo Scientific, Austin, TX, USA) with a TG-5MS direct capillary column (30 m × 0.25 mm × 0.25 µm film thickness). The column oven temperature was initially held at 60°C; this was increased by 6°C/min to 250°C, with a 1-min hold, and then increased to 300°C at 30°C/min. The temperature of the injector was kept at 270°C, with helium as a carrier gas at a flow rate of 1 ml/min. Samples of 1 µl were injected automatically using Autosampler AS3000 coupled with GC. Electron ionization (EI) mass spectra were collected at 70 eV ionization voltage over a range of *m*/*z* 50–650 in full scan mode. The chemical components were identified based on their mass spectra with reference to the spectral databases of the Wiley 09 and NIST14 libraries.

## Results and discussion

### Anatomical study

#### Leaves

The anatomical features of *L. europaeum*, *L. shawii*, and *L. schweinfurthii* var. *aschersonii* were recorded for the first time in this study. Dorsiventral leaves are common in members of the Solanaceae family, but isobilateral leaves are found in some species (Metcalfe and Chalk, 1950; Metcalfe and Chalk, 1979). In the present study, the leaves of the investigated *Lycium* species were found to be isobilateral, where the mesophyll was not clearly differentiated into palisade and spongy parenchyma.

#### Midrib region

The epidermis consisted of a single layer of cells covered by a thick-walled and warty cuticle layer. The epidermis of *L. europaeum* and *L. shawii* was composed of radially elongated cells, while that of *L. schweinfurthii* var. *aschersonii* was composed of radial to tangential cells. Cutin thickness in the upper epidermal cells varied between 6.5 and 8.5 µm in *L. europaeum*, 5.2–6.7 µm in *L. shawii*, and 5–6.1 µm in *L. schweinfurthii* var. *aschersonii*. The thickness of the cutin in the lower epidermal cells ranged between 7.7 and 9.3 µm in *L. europaeum*, 7.2–9.2 µm in *L. shawii*, and 4.5–5.6 µm in *L. schweinfurthii* var. *aschersonii* (**Table 3**).

**Table 3:** Anatomical characteristics of the leaf of the studied *Lycium* species.

Characteristics			*Lycium europaeum*	*Lycium shawii*	*Lycium schweinfurthii* var. *aschersonii*
Leaf anatomy	Midrib	Epidermal layer	Single	Single	Single
		Cuticle thickness	Thick	Thick	Thick
		Upper cuticle thickness (µm)	6.5–8.5	5.2–6.7	5–6.1
		Lower cuticle thickness (µm)	7.7–9.3	7.2–9.2	4.5–5.6
		Cuticle surface	Warty	Warty	Warty
		Epidermis	Radial	Radial	Radial and tangential
		Collenchyma	1 layer	1–2 layers	1–2 layers
		Shape of vascular bundles	Ovate	Ovate	Ovate
		No. of vascular bundles	5	3	5
		Bundle sheath	Parenchyma	Parenchyma	Parenchyma
		Palisade tissue	Discontinuous	Discontinuous	Discontinuous
	Wing	Epidermis	Radial and tangential	Radial	Radial and tangential
		Cuticle thickness	Thick	Thick	Thick
		Cuticle surface	Warty	Warty	Warty
		Type of mesophyll	Isobilateral	Isobilateral	Isobilateral
		Spongy tissue	2 layers	2 layers	2 layers
		No. of lateral vascular bundles	8–9	8–10	11–12
	Crystal druses		Present	Present	Present

**Figure 3:**
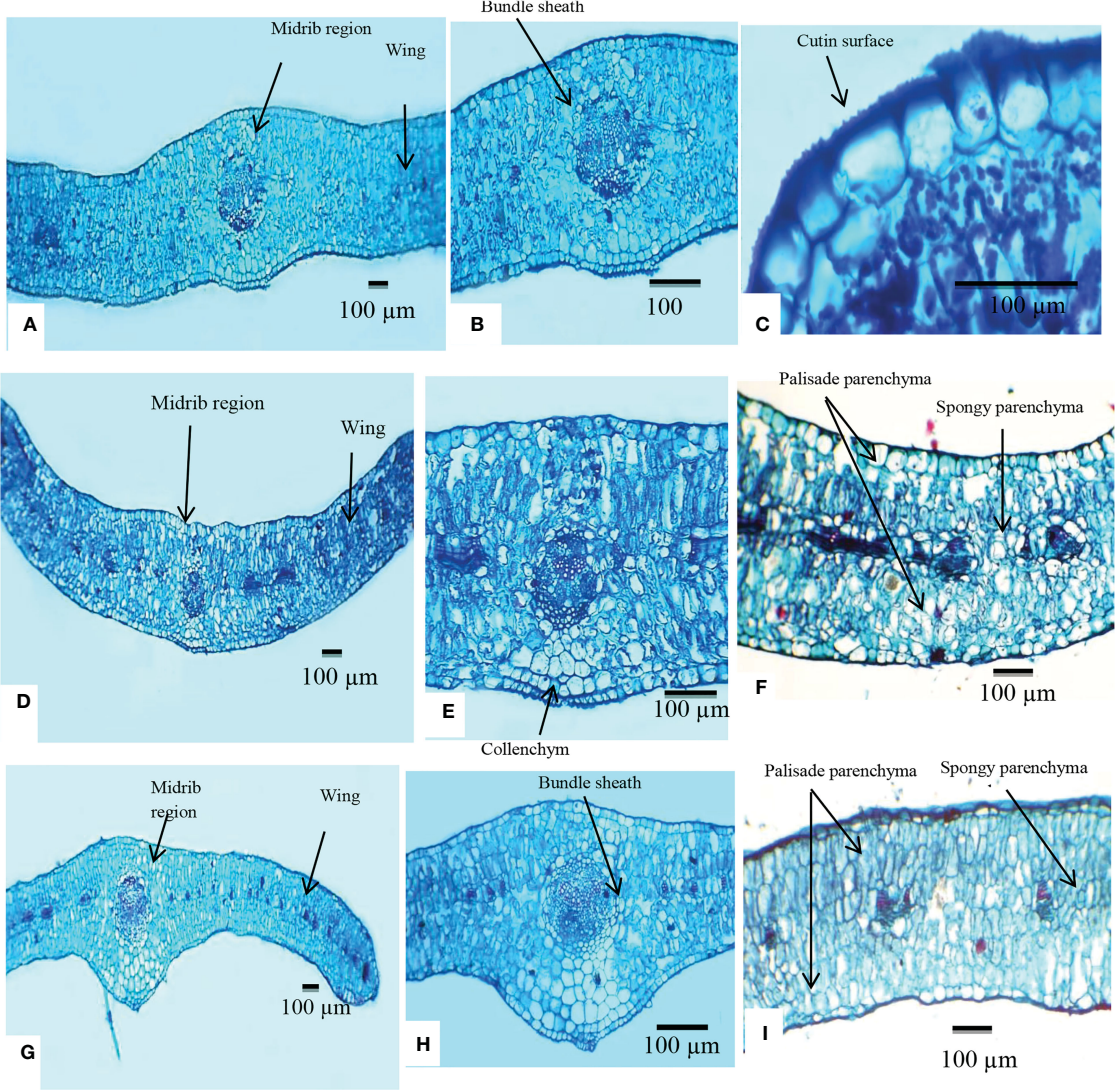
Transverse section of the lamina of *Lycium* species examined using a light microscope. **(A–C)** *Lycium europaeum*. **(D–F)** *Lycium shawii*. **(G–I)** *Lycium schweinfurthii* var. *aschersonii*.

The collenchymatous tissues were recorded as one layer in the adaxial and abaxial surfaces of the epidermis in *L. europaeum* and as one to two layers in *L. shawii* and *L. schweinfurthii* var. *aschersonii*. The midvein consisted of five bicollateral vascular bundles enclosed by a parenchymatous bundle sheath in *L. europaeum* and *L. schweinfurthii* var. *aschersonii*, and three bicollateral vascular bundles enclosed by a parenchymatous bundle sheath in *L. shawii*. In all studied species, the shape of the vascular bundles was ovate (**Table 3** and **Figure 3**).

#### Wing

In *L. europaeum* and *L. schweinfurthii* var. *aschersonii*, the epidermis was represented by a single row of cells with a thick-walled and warty cuticle layer and was composed of both radially elongated and tangential cells, but this layer was composed of only radially elongated cells in *L. shawii*. The mesophyll in the three studied species of *Lycium* was of the isobilateral type, which was represented by palisade cells located under the upper and lower epidermis of the leaf mesophyll. The palisade parenchyma cells were large and elongated, or columnar, and consisted of two to three rows of cells. Spongy parenchyma cells were two-layered. The number of lateral vascular bundles ranged between 8 and 10 in *L. europaeum* and *L. shawii*, but between 11 and 12 in *L. schweinfurthii* var. *aschersonii*. Crystal druses were present in all three studied species (**Table 3** and **Figure 3**).

### Molecular identification of the studied plants

#### Internal transcribed spacer analysis

Sequences of the ITS1–5.8S–ITS2 regions have been recently recognized as being among the universal molecular phylogenetic markers that can be used for plant barcoding, for differentiation of various plant species (Alvarez and Wendel, 2003), and for authentication of interdependent plant species (Jorgenson and Cluster, 1988). Plant genomic DNA was used as a PCR template for the amplification of the ITS regions. From the PCR amplicons, the DNA size was found to be ~700 bp, and the amplicons were sequenced and non-redundantly BLAST-searched in the NCBI database.

**Figure 4:**
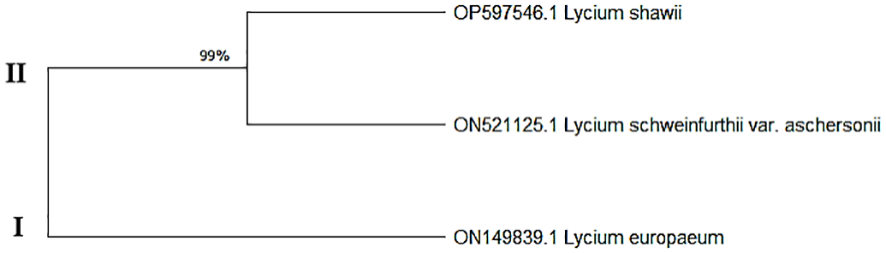
Maximum composite likelihood (MCL) based on the entire internal transcribed spacer (ITS) sequences (ITS5, 5.8S, and ITS6) of the ribosomal DNA of the studied *Lycium* species.

Data on the molecular characteristics (ITS) showed that the studied species belonged to two main clusters in the dendrogram produced by using the ClustalW module of Mega X to execute multiple sequence alignment. Cluster I consisted of one species, *L. europaeum*; cluster II contained two species, *L. shawii* and *L. schweinfurthii* (**Figure 4**).

**Figure 5:**
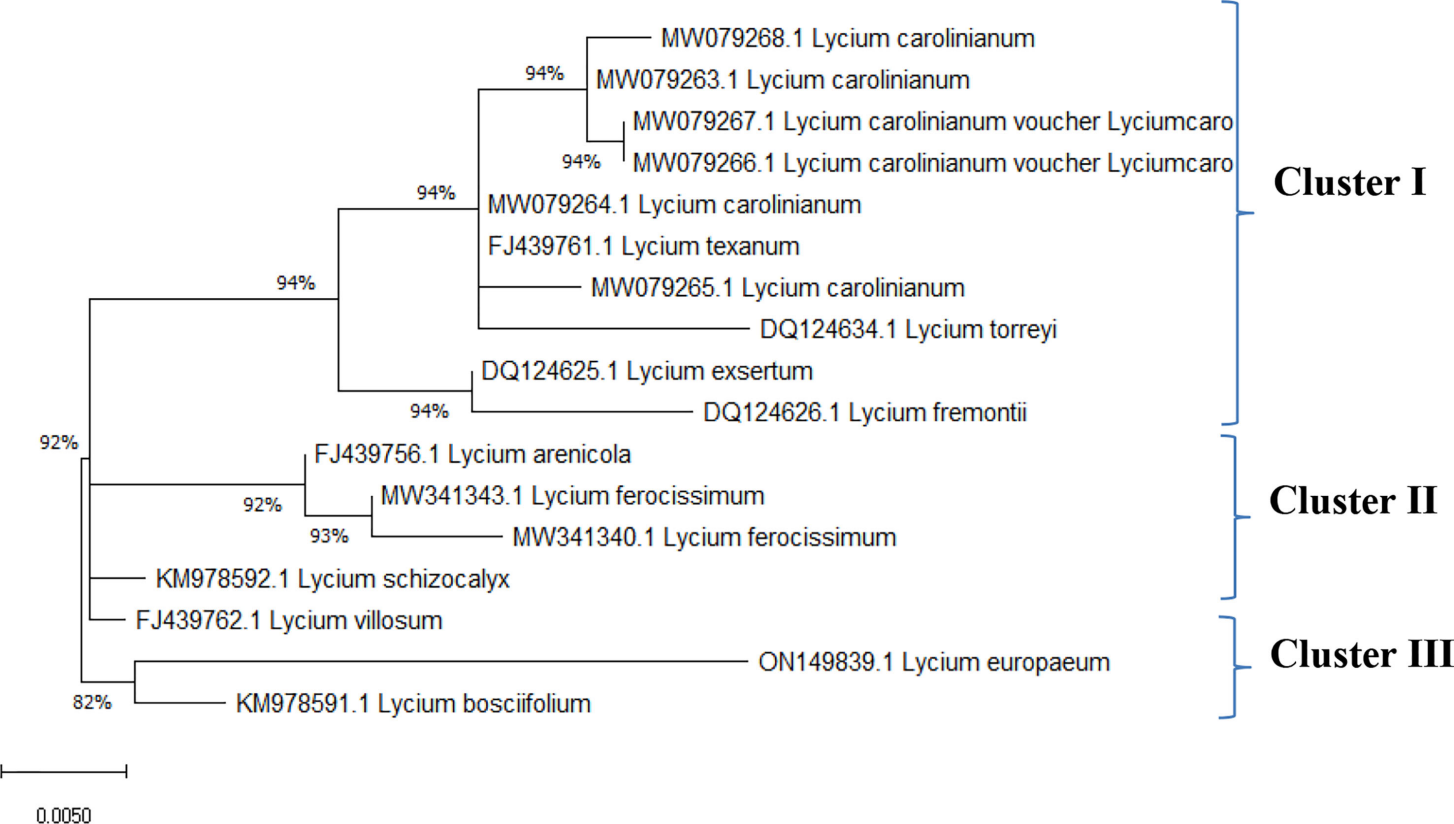
Phylogenetic tree of *Lycium europaeum* compared to database-deposited *Lycium* species in GenBank.

**Figure 6:**
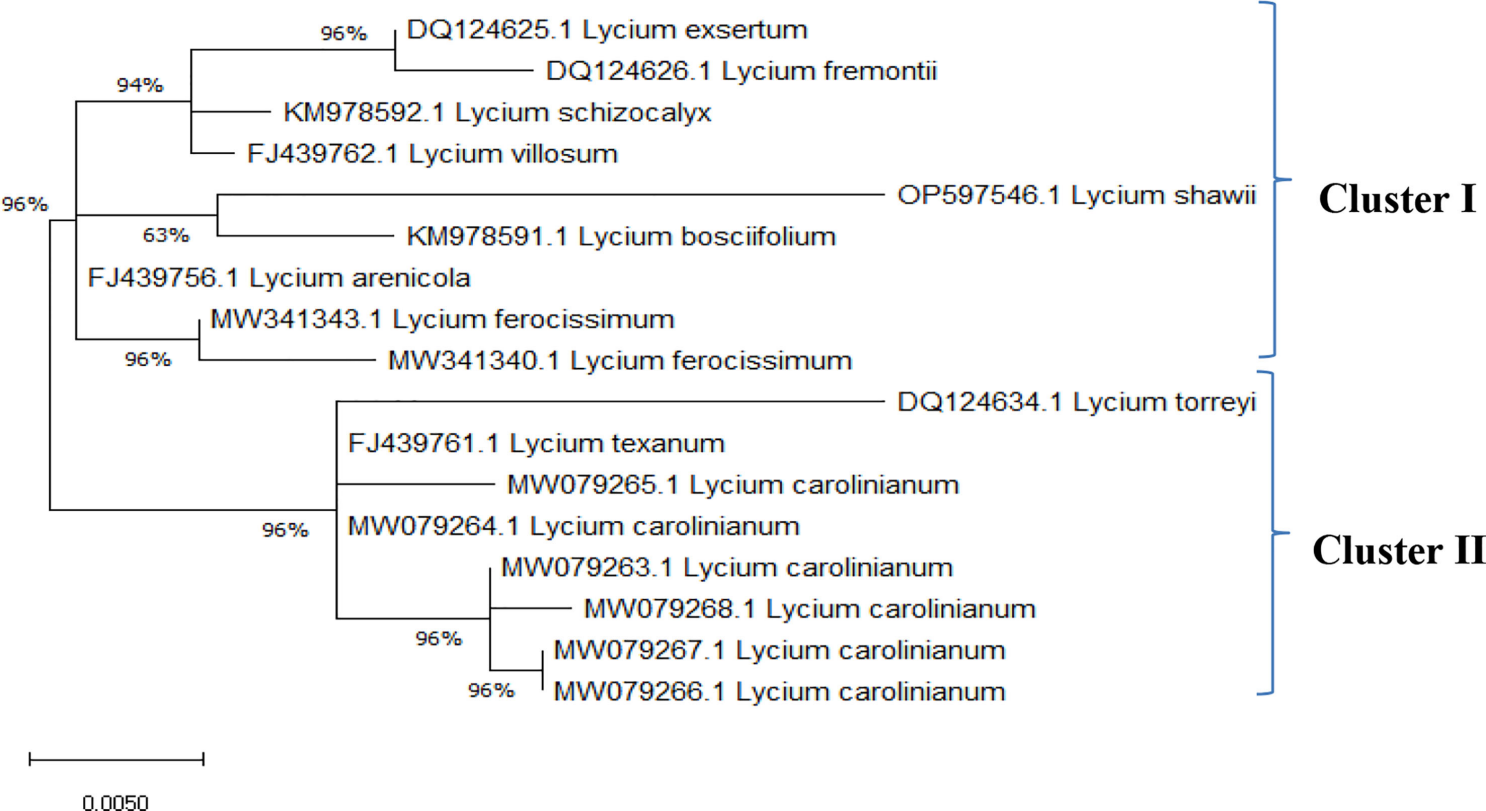
Phylogenetic tree of *Lycium shawii* compared to database-deposited *Lycium* species in GenBank.

**Figure 7:**
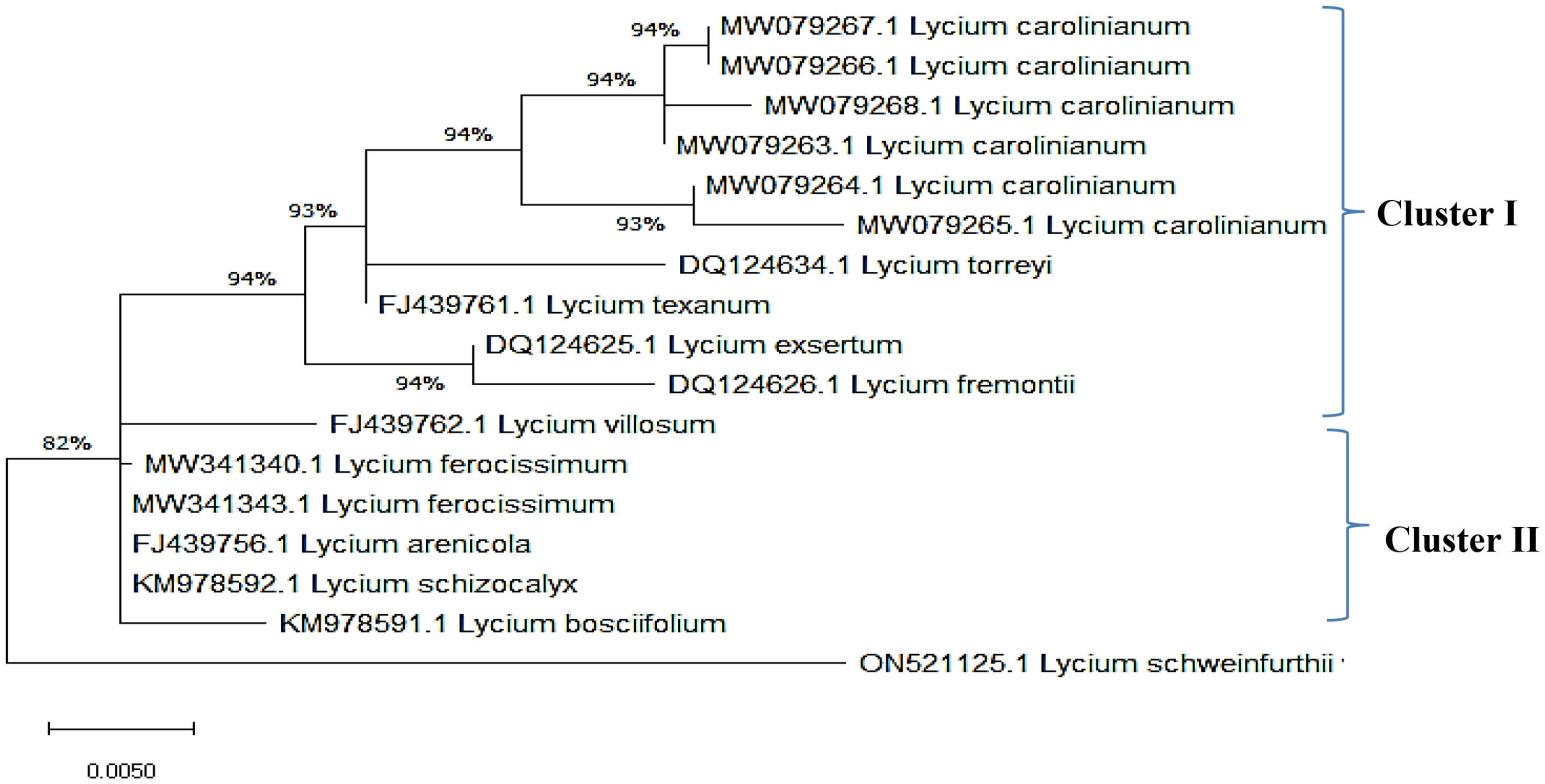
Phylogenetic tree of *Lycium schweinfurthii* var. *aschersonii* compared to database-deposited *Lycium* species in GenBank.

The ITS sequences of *L. europaeum*, *L. shawii*, and *L. schweinfurthii* var. *aschersonii* were deposited into GenBank for the first time with accession numbers ON149839.1, OP597546.1, and ON521125.1, respectively. Phylogenetic analysis of the ITS sequence of *L. europaeum* (ON149839.1) produced three main clusters (clusters I, II, and III) based on the GenBank database (**Figure 5**). *L. europaeum* was mainly found to belong to cluster III, showing 82% similarity to *Lycium bosciifolium* KM978591.1 and 92% similarity to *Lycium ferocissimum* MW3413401.1 and MW341343.1 (cluster II); *Lycium carolinianum* MW079268.1, MW079263.1, MW079267.1, MW079264.1, and MW079265.1; and *Lycium fremontii* DQ124626.1 (cluster I). In contrast, phylogenetic analysis of the ITS sequence of *L. shawii* resulted in only two clusters, where *L. shawii* OP597546.1 exhibited 96% similarity to *Lycium exsertum* DQ124625.1, *Lycium fremootii* DQ124626.1, and *L. ferocissimum* MW341340.1 and MW341343.1 (cluster I), as well as *L. carolinianum* MW079265.1, MW079264.1, MW079268.1, MW0779267.1, and MW079266.1 (cluster II) (**Figure 6**). Similarly, phylogenetic analysis of the ITS sequence of *L. schweinfurthii* produced two clusters. *L. schweinfurthii* ON21125.1 showed 82% similarity to *Lycium ferocissium* MW341340.1 and MW341343.1, *Lycium arenicola* FJ439756.1, and *L. bosciifolium* KM97859.1 (cluster II) (**Figure 7**).

The sequences of the ITS region of these *Lycium* species have not been deposited in GenBank before; therefore, these results are the first records confirming the taxonomic features of these plants using molecular tools based on the ITS sequence region. Based on the phylogenetic relatedness data of the studied *Lycium* species—*L. europaeum*, *L. shawii*, and *L. schweinfurthii*—with the database-deposited species of *Lycium*, three phylogenetic clusters emerged (clusters I, II, and III), where the ITS of the three studied plants belonging to the same cluster (cluster III) showed approximately 61% similarity. The three studied *Lycium* species (cluster III) displayed 93% similarity to *Lycium villosum* FJ439762.1, *L. bosciifolium* MK978592.1, *L. arenicola* FJ439756.1, and *L. ferocissimum* MW341343.1 (cluster II). These species also exhibited 93% similarity to *L. carolinianum* MW079267.1, MW079266.1, MW079263.1, and MW079265.1. DNA barcoding has been recognized as an important approach in molecular systematics, emphasizing the diversity of groups in terms of taxonomic features, due to its independence from environmental and geographical conditions, its time-saving nature, and its feasibility in handling of a very large number of plants. The feasibility of DNA barcoding for molecular taxonomy has been recently validated, especially with the revolution in metagenomics and next-generation high-throughput sequencing technologies, achieving a high degree of accuracy and reproducibility (Huang et al., 2015). Traditional morphometric taxonomy is labor-intensive and time-consuming because it is dependent on the organism’s developmental stage (Costion et al., 2011; Huang et al., 2015). Thus, integrative approaches for plant taxonomic studies have become critical technologies for plant identification, especially given the scientific reductionism of traditional taxonomy (Crisci et al., 2020).

#### Length variation, GC content, nucleotide composition, and mutations of the ITS region

The obtained ITS sequences exhibited differences in the GC content of *L. europaeum*, *L. shawii*, and *L. schweinfurthii* var. *aschersonii* (**Table 4**). The size of the ITS sequences ranged from 444 to 716 bp in *L. shawii* MD2 and *L. europaeum* MD1, respectively. GC content in the studied *Lycium* species was 61.5% in *L. shawii*, 63.6% in *L. europaeum*, and 63.55% in *L. schweinfurthii* var. *aschersonii*. **Table 5** shows the rates of the different transitional substitutions compared to normal transversional substitutions. Nucleotide frequencies were 18.48% (A), 19.17% (T/U), 31.85% (C), and 30.50% (G), and the transition/transversion rate ratios were *k*_1_ = 3.588 (purines) and *k*_2_ = 1.643 (pyrimidines). The overall transition/transversion bias was *R* = 1.211, where *R* = [A*G*k_1_ + T*C*k_2_]/[(A + G)*(T + C)]. All ambiguous positions were removed for each sequence pair (pairwise deletion option), which left a total of 716 positions in the final dataset (**Table 5**). Zhang et al. (2022) performed direct sequencing, genomic DNA cloning, and cDNA cloning to obtain all ITS sequences of six species and two variants of *Lycium* that were not included in the present study. They found that the length of the entire ITS region varied from 532 to 691 bp with the primer pair ITS1/ITS4, with an alignment of 729 bp. The average GC content of the ITS1, 5.8S, and ITS2 putative pseudogenes was 47.9% and 43.4, 46.1% and 47.1%, and 50.8% and 52.6%, respectively. In a study on *Quercus* spp., the nucleotide composition frequencies were 20.86, 18.83, 29.65, and 30.67% for A, T, C, and G, respectively (Bellarosa et al., 2005). Sharma et al. (2002) reported that the ITS regions of wheat and barley varied between 597–605 bp and 595–598 bp, respectively. In contrast, it was found that the average nucleotide frequencies of the ITS region among different taxa of the Asteraceae family were 25% (A), 24% (T), 26% (C), and 25% (G) (Amar et al., 2012). The overall length of the ITS region of *Ficus carica* (family Moraceae) was found to be 697.5 bp, while the composition of the bases was 19.7% (A), 18.6% (T), 31.4% (C), and 30.2% (G) (Baraket et al., 2013). After analysis of the ITS sequence of Naga King chili, it was found that the sequence exhibited nucleotide frequencies of 18.85%, 17.56%, 33.95%, and 29.64% for A, T, C, and G, respectively, while the average size of the ITS region in the plant was 620 bp (Kehie et al., 2016). In our previous study on the ITS region of species of *Cassia* and *Senna*, it was found that the average size of the ITS region varied from 403 to 796 bp, while the detected nucleotide frequencies were 20.86% (A), 18.83% (T/U), 29.65% (C), and 30.67% (G). On the intergenic spacer ITS sequences, transitions were more common than transversions (Eldemerdash et al., 2022). However, older studies have proposed that the size of the ITS region in angiosperms ranges from 565 to 700 bp (Liston et al., 1996).

**Table 4:** Length and GC content of the ribosomal DNA sequences of the studied taxa.

Scientific name	Accession number	Length (bp)	GC content (%)
*Lycium europaeum*	ON149839	716	63.68
*Lycium shawii*	OP597546	444	61.53
*Lycium schweinfurthii* var. *aschersonii*	ON521125	708	63.55

**Table 5:** Maximum composite likelihood estimation of the pattern of nucleotide substitution of *Lycium* species.

	A	T	C	G
**A**	–	4.17	6.93	**23.81**
**T**	4.02	–	**11.39**	6.64
**C**	4.02	**6.85**	–	6.64
**G**	**14.43**	4.17	6.93	–

Each entry shows the probability of substitution (r) from one base (row) to another base (column). Evolutionary analyses were conducted in MEGA X (Kumar et al., 2018).

#### Start codon target analysis

**Figure 8:**
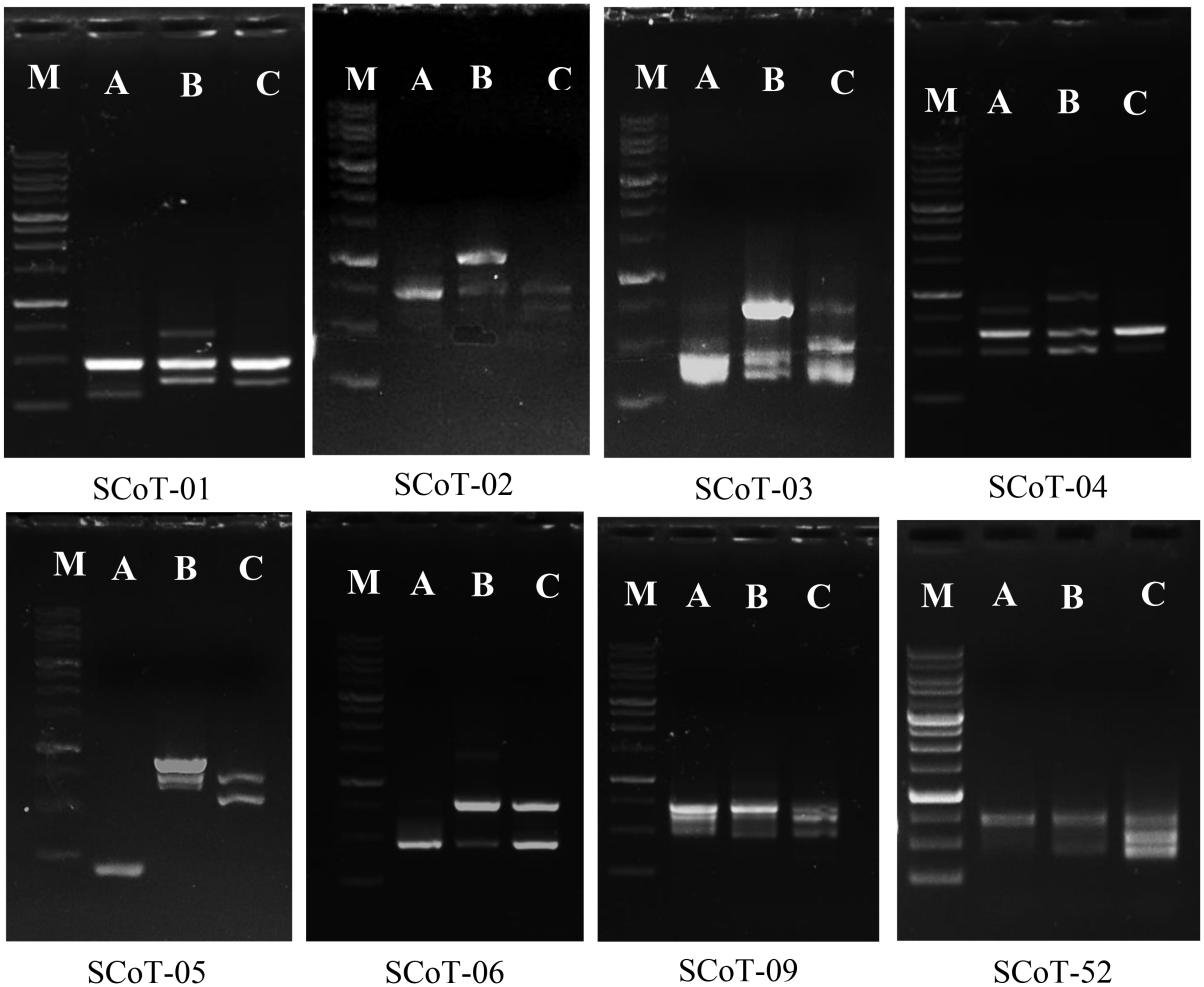
Band profiling of start codon targeted (SCoT) markers for *Lycium* species. *M*, marker 1-kb DNA ladder. **(A)** *Lycium europaeum*. **(B)** *Lycium shawii*. **(C)** *Lycium schweinfurthii* var. *aschersonii*.

The amplification of specific sequences of *L. europaeum*, *L. shawii*, and *L. schweinfurthii* var. *aschersonii* was analyzed using eight selected SCoT primers (**Table 6** and **Figure 8**). In total, there were 62 fragments amplified by all primers in the studied species, including 44 polymorphic fragments with a ratio of 70.97%. The highest polymorphism between species, of 90%, was observed with SCoT-09 primer, in which nine polymorphic primers were detected. The lowest polymorphism, of 40%, and the lowest number of amplicons (only five amplified fragments) were obtained with SCoT-05 primer. Five of the eight primers showed polymorphism over 71%. All SCoT primers produced unique fragments in the studied species. The highest number of unique amplicons, with 11 fragments, was observed in *L. shawii*, while five and four amplicons were obtained in *L. europaeum* and *L. schweinfurthii* var. *aschersonii*, respectively. Eight unique negative markers were obtained in the studied genomes: four in *L. shawii*, three in *L. europaeum*, and one *L. schweinfurthii* var. *aschersonii*.

**Table 6:** Amplification and polymorphism frequency based on start codon targeted (SCoT) analysis of *Lycium* species.

Primers	Total no. of amplicons	Monomorphic amplicons	Polymorphic amplicons	Percentage of polymorphism
SCoT-01	7	2	5	71.4
SCoT-02	9	2	7	77.7
SCoT-03	8	2	6	75
SCoT-04	8	3	5	62.5
SCoT-05	5	3	2	40
SCoT-06	8	2	6	75
SCoT-09	10	1	9	90
SCoT-52	7	3	4	57.1
Average	7.75	2.25	5.5	68.5

### GC-MS metabolic profiling analysis

Metabolic profiling of the studied plants was conducted as a marker for authentication of the traditional taxonomic features and DNA barcoding analysis (Eldemerdash et al., 2022). Estimation of volatile chemical compounds by GC-MS for plant differentiation has been recognized as one of the key technological tools for metabolic profiling and taxonomic analysis that can be used to confirm traditional taxonomic features. Traditional morphometric taxonomy is time-consuming due to its dependence on the plant’s developmental stage and involves labor-intensive work based on predetermined categories (Costion et al., 2011; Huang et al., 2015). GC-MS metabolic profiling has been frequently used for taxonomic purposes in Convolvulaceae (Schimming et al., 2005), the tree fern *Cyathea* (Cyatheaceae) (Janakiraman and Johnson, 2016), *Solanum* spp. (El-Shaboury et al., 2017), *Centaurea galicicae* and *Centaurea tomorosii* (Asteraceae) (Janaćković et al., 2017; Wang et al., 2018), and in seven taxa of the subfamily Caesalpinioideae (Eldemerdash et al., 2022). GC-MS has also been used in the detection of bioactive constituents of plants, which might make it extremely useful for drug research and discovery (Janakiraman et al., 2012; Rukshana et al., 2017).

**Figure 9:**
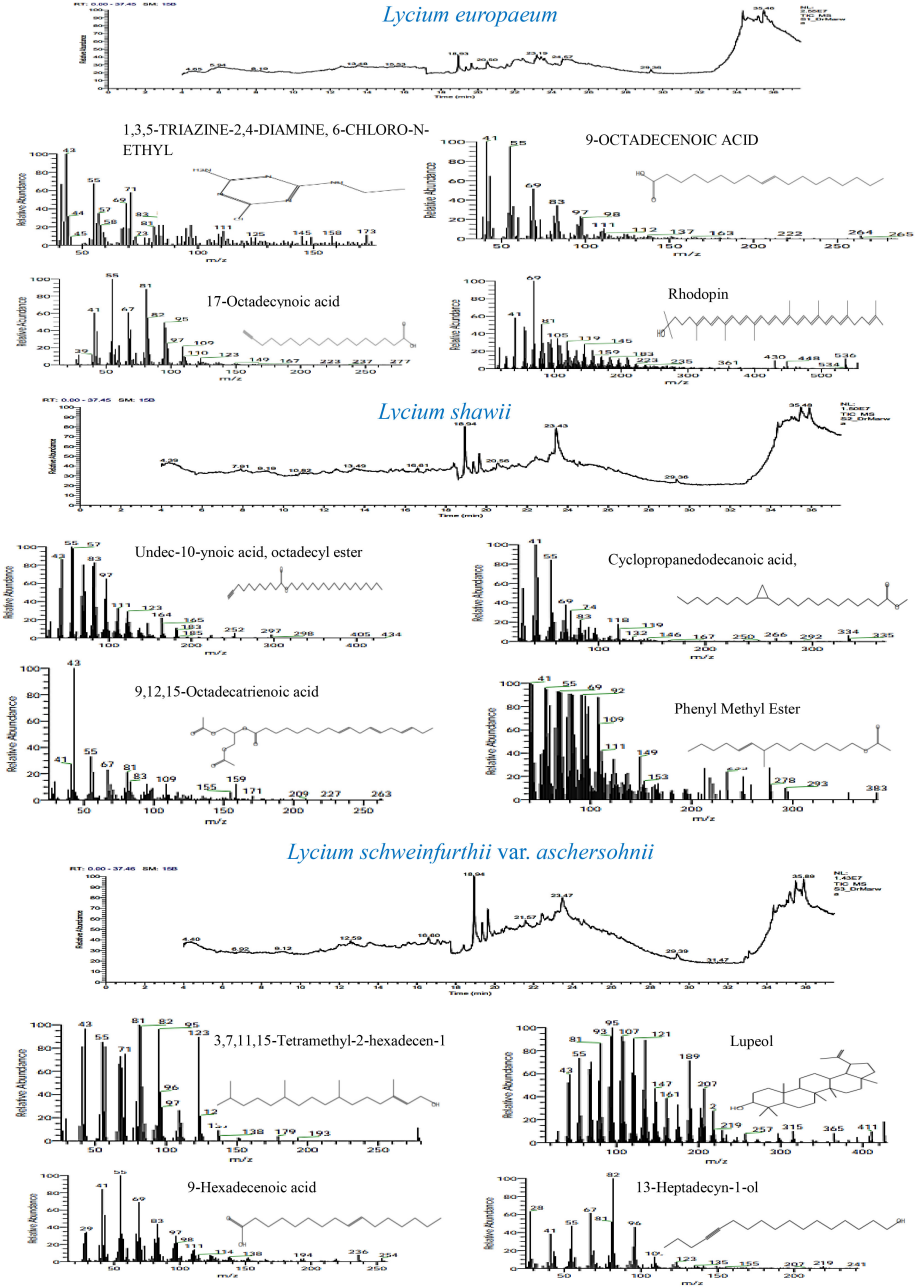
GC-MS chromatograms of the methanol leaf extract of *Lycium* species.

GC-MS total ion current chromatograms of the extracts of the studied taxa of *Lycium* species are shown in **Figure 9**. Phytochemical compounds were identified in the methanol extracts of the leaves examined using GC-MS analysis; the most prevalent of these in the extracts are represented. Compounds with corresponding peaks were identified in the studied species. The retention time, molecular formula, molecular weight, chemical class, and concentration percentage (area percentage) of the identified compounds were also determined. Metabolic profiling of the studied species identified 34 compounds with obvious fluctuations in their ethanolic extracts. Of these, three major compounds were shared between the plant species (**Table 7**). The results revealed clear variation in the percentage (area percentage) of the observed compounds between the studied taxa, as shown by the area of the peaks in the chromatogram. The compounds 1,3,5-triazine-2,4-diamine, 13-heptadecyn-1-ol, and 1-heptatria-cotanol consistently occurred among the studied species. In addition, several of the identified phyto-compounds were designated as exclusive diagnostic chemical traits of individual taxa. For instance, 9-octadecenoic acid-isochiapin, linoleic acid ethyl ester, palmitic acid, 17-octadecynoic acid, rhodopsin, and betulin were assigned to *L. europaeum.* These compounds have anti-inflammatory, anti-ulcer, antidiabetic, antibacterial, antimicrobial, antimalarial, antiviral, anti-hyperlipidemic, anticancer, and anti-HIV properties (Aparna et al., 2012; Yao et al., 2013; Boparai et al., 2017; Lan et al., 2017). In contrast, 9,12,15-octadecatrienoic acid, 4-hydroxy-, methyl ester, undec-10-ynoic acid, octadecyl ester, cyclopropanedodecanoic acid, 2-octyl, oxiraneundecanoic acid, and phenylmethyl ester were diagnostic for *L. shawii.* In a recent study, the compounds isolated from *L. shawii* were examined for their anticancer potential in breast cancer cell lines. The results showed that some of these compounds exhibit excellent anti-proliferative activity (Ur Rehman et al., 2020).

**Table 7:** GC-MS metabolic profiling of the studied *Lycium* species.

No.	RT (min)	Compound name	Area (%)			RI
			*L. europaeum*	*L. shawii*	*L. schweinfurthii*	
1	4.03	2-Acetyl-3-(2-benzenesulphonamido)ethyl-7-methoxyindole	–	1.02	1.22	875
2	13.48	Ethanimidothioic acid	0.91	–	–	2,041
3	13.49	9,12,15-Octadecatrienoic acid	–	1.59	–	2,077
4	15.88	2-(7-Heptadecynyloxy) tetrahydro	–	1.74	–	2,453
5	16.61	6-Chloro-*n*-ethyl	–	2.05	2.28	1,699
6	17.17	9-Octadecenoic acid	4.31	–	–	2,175
7	18.41	1,3,5-Triazine-2,4-diamine, 4-chloro-*n*-ethyl	1.01	6.90	3.27	1,285
8	18.55	Undec-10-ynoic acid, octadecyl ester	–	3.14	–	2,019
9	18.93	17-Octadecynoic acid	7.51	–	–	2,165
10	18.94	3,7,11,15-Tetramethyl-2-hexadecen-1-ol	–	16.34	20.31	2,192
11	19.35	Linoleic acid ethyl ester	2.19	–	–	2,193
12	19.65	13-Heptadecyn-1-ol	3.06	8.63	11.52	1,971
13	19.96	1,25-Dihydroxyvitamin	0.97	–	–	3,150
14	19.99	Picrotoxin	–	0.89	–	2,068
16	20.49	13,16-Octadecadiynoic acid	4.17	2.25	–	2,112
17	20.55	Oxiraneundecanoic acid	–	2.94	–	2,129
18	21.57	Palmitic acid	2.08	–	–	1,968
19	21.57	1-Heptatriacotanol	2.54	7.67	6.09	3,942
20	23.10	Cyclododecanepentanoic acid	1.71	1.80	–	1,944
21	23.10-23.21	9-Hexadecenoic acid	–	2.25	1.69	1,976
22	23.19	2-(9-Octadecenyloxy)	3.59	–	–	2,640
23	23.43	Phenylmethyl ester	–	10.16	–	1,284
24	23.47	Aspidospermidin-17-ol	–	–	5.39	2,942
25	24.32	Trideuteriomethyl 10-epoxy-7-ethyl-3,11-dimethyltrideca-2,6-dienoate	–	1.23	1.88	2,025
26	24.57	11,14-Eicosadienoic acid, methyl ester	–	1.58	–	2,638
27	24.58	9,12-Octadecadienoyl chloride	4.74	–	–	2,139
28	24.76	9,12-Octadecadienoyl chloride, (*Z*,*Z*)-	2.71	–	–	2,139
29	29.36	4h-1-Benzopyran-4-1,5,7-dihydroxy-2-(4-hydroxyphenyl)-3-methoxy	2.12	–	–	3,749
30	34.33	Rhodopin	9.85	–	–	4,025
31	34.34	1-Heptatriacotanol	–	3.54	3.50	3,942
32	34.66	Carotene, 1,1′,2,2′-tetrahydro-1,1′-dimethoxy	0.99	–	1.18	3,978
33	34.71	3-Ethyl-3-hydroxy-	1.58	–	–	1,144
34	35.18	9,10-Secocholesta-5,7,10(19)-triene-3,24,25-triol, (3á,5*Z*,7*E*)-	3.54	–	4.17	2,806
35	35.46	Androstan-17-1, 3-ethyl-3-hydroxy	11.09	–	–	2,707
36	35.49	Stigmast-5-en-3-ol, (3á,24S)-	–	7.67	6.09	2,731
37	35.88	Betulin	2.01	–	–	3,090
38	35.89	Lupeol	–	–	6.29	2,848

RT, retention time; RI, retention index.

The absence of some phyto-compounds in certain species might be considered a chemical taxonomic feature for specific species compared to the other two species. For example, the absence of 3,7,11,15-tetramethyl-2-hexadecen-1-ol may be a characteristic of *L. europaeum*. Moreover, the absence of cyclododecanepentanoic acid may be a characteristic of *L. schweinfurthii* var. *aschersonii*. The presence of aspidospermid-17-ol and lupeol represents a diagnostic metabolomics tool for *L. schweinfurthii* var. *aschersonii.* Lupeol has been found in a preclinical study to be a therapeutic and chemopreventive agent with anti-inflammatory and anticancer activities (Saleem, 2009).

The presence and frequencies of the shared metabolites revealed common characteristics and metabolic identifiers for *Lycium* species. *L. europaeum* is characterized by the presence of numerous secondary metabolites, including androstan-17-1, 3-ethyl-3-hydroxy (11%), rhodopin (9.8%), 13,16-octadecadiynoic acid (4.2%), 9-octadecanoic acid (4.3%), and ethanimidothioic acid (0.9%). The unique frequencies of these compounds revealed the distinctive metabolic pattern of *L. europaeum* compared to other *Lycium* species. The compound 3,7,11,15-tetramethyl-2-hexadecen-1-ol was recorded in *L. schweinfurthii* (20.3%) and *L. shawii* (16.4%), but was absent in *L. europaeum*. The compound 13-heptadecyn-1-ol was reported in different concentrations among the *Lycium* species: at 3.1% in *L. europaeum*, 8.6% in *L. shawii*, and 11.5% in *L. schweinfurthii*. The highest concentration of the shared metabolite l-heptatriacotanol was reported in *L. shawii* (7.67%), followed by *L. schweinfurthii* (6.1%) and then *L. europaeum* (2.5%), revealing the frequency of this metabolite as species-dependent. Lupeol was mainly detected in the extract of *L. schweinfurthii* (6.25%), and was completely absent in *L. europaeum* and *L. shawii*. Thus, metabolic profiling of the studied *Lycium* species could be a distinguishing taxonomic key, revealing the role of chemical constituents in identifying species.

Several distinctive metabolites were assessed using a chemotaxonomic marker/identifier for each species of *Lycium*. Of the 32 phytochemical compounds identified, several were demonstrated to be unique chemical traits for individual species. In addition to taxonomic features, these metabolites have also been reported to possess antimicrobial, antioxidant, anti-inflammatory, anticancer, antidiabetic, and anti-arthritic activities, as well as can be used in food preservation, degreasing, perfume production, wound dressing, and in cosmetics. During the past two decades, there has been an increase in the screening of chemical compounds using GC-MS analysis for use as chemical markers in the classification of plants (Salimpour et al., 2011; El-Shaboury et al., 2017; Wang et al., 2018; Eldemerdash et al., 2022) or the discovery of therapeutic agents (Powder-George and Mohamed, 2018; Selvaraj et al., 2020). Information on these phytochemicals can serve as a starting point, especially in research on and discovery of novel drugs and other bioactive metabolites for industrial applications (Misra and Srivastava, 2016). Chemotaxonomy has received much attention as one of the most effective methods for the assessment of the biological variety of plants, highlighting their morphological and genetic diversity and clarifying their evolutionary implications for a specific taxon (Gottlieb, 1982).

Chemotaxonomic features exhibit a distinct gene expression paradigm concerning environmental variables, resulting in the identification of distinct chemotaxonomic indicators for every taxon (Santos et al., 2010; Ali et al., 2016). The existence of specific metabolites, i.e., “phyto-markers,” in a particular taxon might greatly simplify the metabolic mining of substances of pharmacological and commercial interest (Oladipo et al., 2017). Phytochemical studies have also indicated that the various biological activities of *Lycium* species are related to their richness in different classes of compounds (e.g., polysaccharides, carotenoids, flavonoids, alkaloids, amides, and terpenoids, among others) (Yao et al., 2011; Qian et al., 2017). Moreover, it has been reported that extracts of plant-based metabolites, especially polysaccharides and vitamins, promote anti-aging, eyesight improvement, and anti-fatigue effects (Wu and Guo, 2015; Yao et al., 2017).

### Numerical analysis

**Figure 10:**
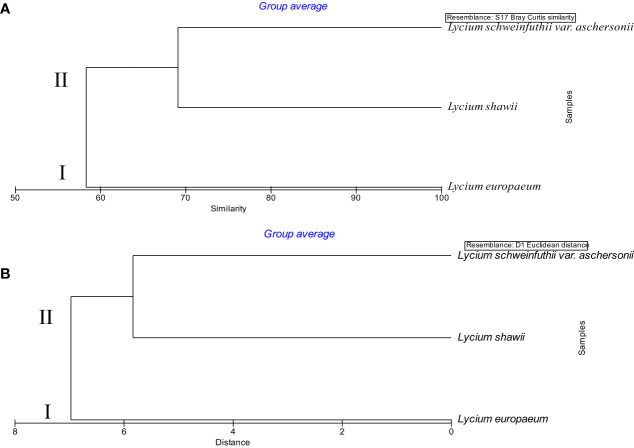
Dendrogram of the relationship between the three taxa of *Lycium* based on combined morphological features, start codon targeted (SCoT) markers, and chemical profiles, determined using PRIMER analysis. **(A)** Similarity. **(B)** Distance.

Anatomical, chemical, and molecular features were analyzed to assess the taxonomic relatedness of the plants studied. The traits of the three *Lycium* species, including 15 leaf anatomical characteristics, 38 chemical features, and 35 SCoT banding patterns, were entered into numerical analysis. A dendrogram, consisting of two main clusters, was generated using PRIMER software. *L. europaeum* was separated in the first cluster at a 58.68% similarity level and a distance of 7.42. The second cluster was composed of *L. shawii* and *L. schweinfurthii* var. *aschersonii* at a 68.15% similarity level and a distance of 6.57 (**Figure 10**). The results of the PRIMER analysis of the combined data on anatomical features, SCoT markers, and chemical profiling were consistent with the results of the ITS markers. This indicates that these features are informative markers for species discrimination and identification. Furthermore, our results are consistent with those of El-Ghamery et al. (2018), showing that *L. shawii* is more closely related to *Lycium schweinfurthii* var. *aschersonii* than to *L. europaeum*.

### Ecological analysis of the studied plants

**Figure 11:**
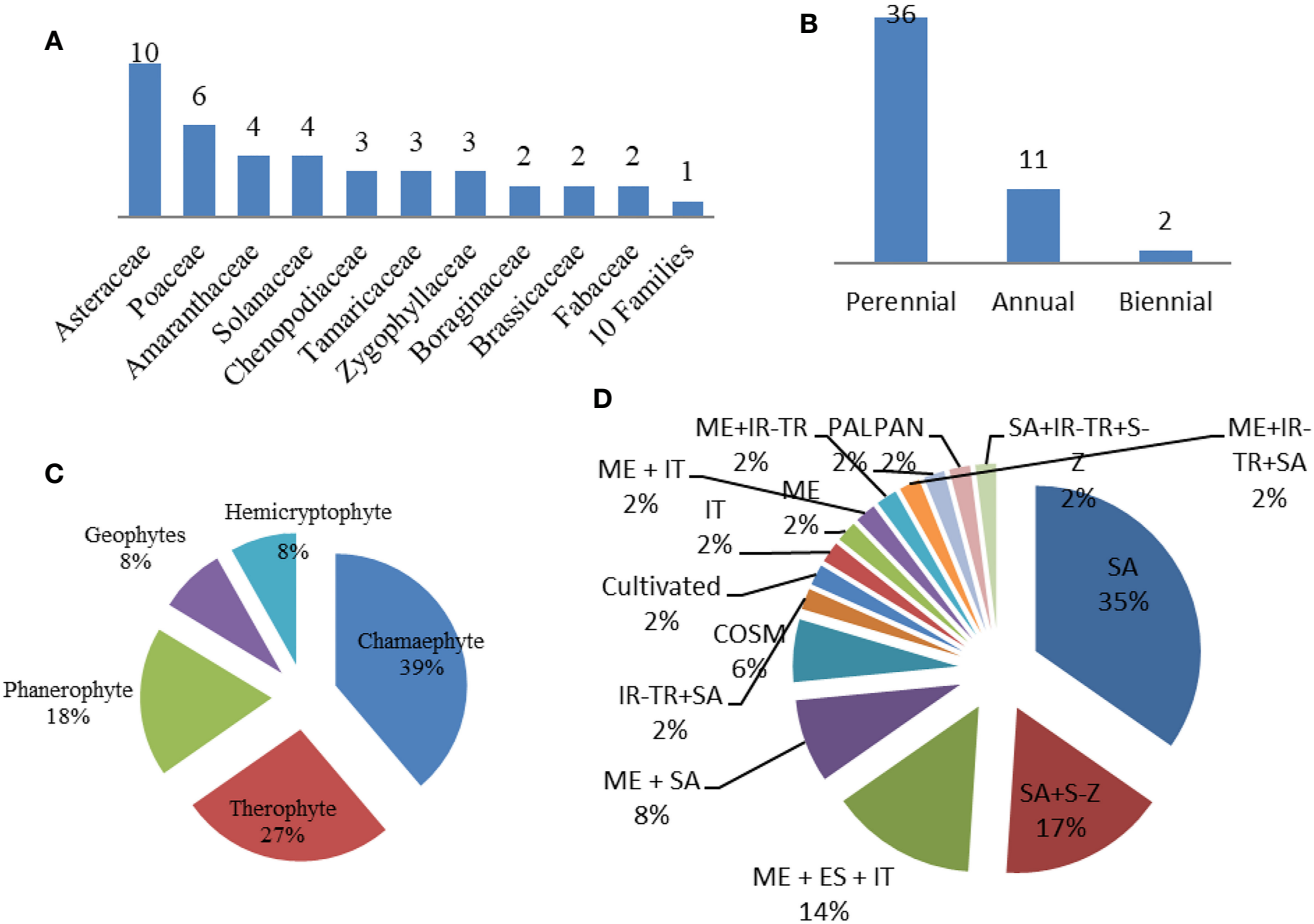
Ecological studies of *Lycium* species. **(A)** Families. **(B)** Life span. **(C)** Life form. **(D)** Floristic category. SA, Saharo–Sindian; S–Z, Sudano–Zambesian; ME, Mediterranean; ES, Siberian; COSM, Cosmopolitan; IT, Irano–Turanian; PAL, Paleotropical; PAN, Pantropical; S–Z, Sudano–Zambesian.

A total of 49 taxa belonging to 20 families were recorded within the plant communities associated with the *Lycium* genus in the studied area (Wadi Hagul, the Mediterranean coast of Egypt, and Alkom Alakhdar Island—El Brullus Lake). Approximately 27 species were recorded exclusively in Hagul. These species have not been recorded in other regions; moreover, seven species were recorded on the Mediterranean coast of Egypt, while 14 species were recorded in Alkom Alakhdar Island. Only a single species, *Deverra tortuosa*, was shared between the areas of Wadi Hagul and the Mediterranean coast of Egypt. Asteraceae and Poaceae were the most frequent families, with 10 and 6 species, respectively. Two families were represented by four species (Amaranthaceae and Solanaceae), while three families were represented by three species (Chenopodiaceae, Tamaricaceae, and Zygophyllaceae). Boraginaceae, Brassicaceae, and Fabaceae were represented by two species each. There were 10 families represented by a single species (**Table 8** and **Figure 11**). Asteraceae was the most prevalent in several previous studies, such as in the Mediterranean North African flora (Quézel, 1978), in Egyptian flora (Täckholm, 1974; Boulos, 2002), and in Wadi Hagul (Marie, 2000; Zahran and Willis, 2009; Abdelaal, 2016; Bedair et al., 2021). Furthermore, Asteraceae is known for having salt-tolerant and xerophytic species (Aronson, 1989).

**Table 8:** Ecological characteristics of recorded species in the plant communities of *Lycium*.

Species	Family	Life span	Life form	Floristic category	Record
*Mesembryanthemum nodiflorum* L.	Aizoaceae	Ann	Th	ME+ES+SA	C
*Atriplex canescens* (Pursh) Nutt.	Amaranthaceae	Per	Ch	ME	C
*Beta vulgaris* L.		Ann	Th	ME+ES+IT	C
*Suaeda vera* Forssk. ex J.F.Gmel.		Per	Ch	ME+ES+SA	C
*Allium roseum* L.	Amaryllidaceae	Per	Geo	SA	C
*Deverra tortuosa* (Desf.) DC.	Apiaceae	Per	Ch	SA	A, B
*Pulicaria undulata* (L.) C.A.Mey.	Asteraceae	Per	Ch	SA+S-Z	A
*Reichardia tingitana* (L.) Roth		Ann	Th	ME+IT	A
*Senecio glaucus* L.		Ann	Th	ME+IT+SA	A
*Echinops spinosissimus* subsp. *spinosissimus*		Per	Hem	ME+SA	A
*Centaurea aegyptiaca* L.		Bi	Th	SA	A
*Iphiona mucronata* (Forssk.) Asch. & Schweinf.		Per	Ch	SA	A
*Launaea nudicaulis* (L.) Hook.f.		Per	Hem	SA	A
*Launaea spinosa* (Forsk.) Sch.Bip. ex Kuntze		Per	Ch	SA	A
*Asteriscus graveolens* (Forssk.) Less.		Per	Ch	SA	A
*Centaurea alexandrina* Delile		Bi	Th	SA	B
*Trichodesma africanum* (L.) Sm.	Boraginaceae	Per	Ch	SA+S-Z	A
*Heliotropium arbainense* Fresen.		Per	Ch	SA	A
*Capsella bursa-pastoris* (L.) Medik.	Brassicaceae	Ann	Th	COSM	C
*Zilla spinosa* (L.) Prant.		Per	Ch	SA	A
*Gymnocarpos decander* Forssk.	Caryophyllaceae	Per	Ch	SA	A
*Arthrocaulon macrostachyum* (Moric.) Piirainen & G.Kadereit	Chenopodiaceae	Per	Ch	ME+SA	C
*Atriplex halimus* L.		Per	Ph	ME+SA	B
*Haloxylon salicornicum* (Moq.) Bunge ex Boiss.		Per	Ch	SA	A
*Convolvulus stachydifolius* Choisy	Convolvulaceae	Per	Hem	IT	B
*Ephedra alata* Decne.	Ephedraceae	Per	Ch	SA	A
*Retama raetam* (Forssk.) Webb& Berthel.	Fabaceae	Per	Ph	SA	A
*Vachellia tortilis* (Forssk.) Galasso & Banfi		Per	Ph	SA+S-Z	A
*Juncus acutus* L.	Juncaceae	Per	Geo	ME+ES+IT	C
*Malva parviflora* L.	Malvaceae	Ann	Th	ME+IT	C
*Avena sativa* L.	Poaceae	Ann	Th	COSM	B
*Phragmites australis* (Cav.) Trin. ex Steud.		Per	Geo	COSM	C
*Hordeum marinum* Huds.		Ann	Th	ME+ES+IT	C
*Setaria viridis* (L.) P.Beauv.		Ann	Th	ME+ES+IT	C
*Cynodon dactylon* (L.) Pers.		Per	Geo	PAN	B
*Panicum turgidum* Forssk.		Per	Hem	SA	A
*Ranunculus sceleratus* L.	Ranunculaceae	Ann	Th	ME+ES+IT	C
*Ochradenus baccatu*s Delile.	Resedaceae	Per	Ph	SA	A
*Kickxia aegyptiaca* (L.) Nábělek	Scrophulariaceae	Per	Ch	ME+SA	A
*Lycium shawii* Roem. & Schult.	Solanaceae	Per	Ph	SA+S-Z	A
*Lycium europaeum* L.		Per	Ph	SA+S-Z	B
*Lycium schweinfurthii* Dammer		Per	Ph	SA+S-Z	C
*Nicotiana glauca* Graham		Per	Ph	Cultivated	B
*Reaumuria hirtella* Jaub. & Spach.	Tamaricaceae	Per	Ch	IT+SA	A
*Tamarix aphylla* (L.)H. Karst.		Per	Ch	SA+IR-TR+S-Z	A
*Tamarix nilotica* (Ehrenb.) Bunge.		Per	Ph	SA+S-Z	A
*Zygophyllum coccineum* L.	Zygophyllaceae	Per	Ch	SA+S-Z	A
*Zygophyllum simplex* L.		Ann	Th	PAL	A
*Fagonia arabica* L.		Per	Ch	SA	A

Ann, annual; Per, perennial; Th, therophyte; Ch, chamaephyte; Ph, phanerophyte; Hem, hemicryptophyte; Geo, geophyte. A: Recorded in Wadi Hagul. B: Recorded in Alexandria—Matruh coastal road. C: Recorded in Alkom Alakhdar Island, El Brullus.

In terms of life span, the recorded species were perennial (36 species), annual (11 species), or biennial (two species) (**Table 8** and **Figure 11**). Life forms of the recorded species included chamaephytes (19 species), therophytes (13 species), phanerophytes (nine species), and geophytes and hemicryptophytes (four species each) (**Table 8** and **Figure 11**). The increase in the number of perennials over annual species may be due to the lack of rainfall in the study areas during the relevant period. Rainfall is one of the most strongly determining factors for life span and life form patterns. Chamaephyte was the most frequent life form, at 39%. This result is largely consistent with several previous studies that were conducted in Wadi Al-Assiuty (Abd El-Galil, 2014) and Wadi Hagul, Eastern Desert, Egypt (Bedair et al., 2021). In semi-arid and arid areas, most of the life forms take the form of chamaephytes (Jafari et al., 2016).

Saharo–Sindian species comprised the most common floristic category (*n* = 17, 34.7%), followed by Saharo–Sindian–Sudano–Zambesian species (*n* = 8, 16.3%), Mediterranean–Euro–Siberian–Irano-Turanian species (*n* = 7, 14.3%), Mediterranean–Saharo–Sindian species (*n* = 4, 8.2%), and Cosmopolitan species (*n* = 3, 6.1%), while 10 phytochoria were represented by a single species (**Table 8** and **Figure 11**). Saharo–Sindian phytochoria comprised the maximum proportion in the floristic category, with more than 34%. These species are good indicators of a desert environmental habitat (Bouallala et al., 2020).

## Conclusions

The present study has demonstrated the similarities and differences between three species of the genus *Lycium* that are widespread in Egypt: *L. europaeum* L., *L. shawii* Roem. & Schult., and *L. schweinfurthii* var. *aschersonii* (Dammer) Feinbrun. The species’ anatomical, ecological, and molecular characteristics, as well as their metabolic profiles, were investigated. The study has presented simple, clear, and sufficiently differentiating characteristics, which could help researchers to distinguish between the three species anatomically. Furthermore, the ecological biodiversity associated with these three species has provided an account of the nature of their ecosystems and their interactions. The studied *Lycium* species were first molecularly identified based on their ITS sequences, which were deposited in GenBank. Moreover, SCoT polymorphism analysis was performed to develop additional criteria for further identification between the species. Regarding the chemical profiling, the compounds 1,3,5-triazine-2,4-diamine, 6-chloro-*N*-ethyl, and 13-heptadecyn-1-ol were consistent among the investigated taxa, while 23 unique chemicals were identified in the studied species (14 compounds in *L. europaeum*, seven compounds in *L. shawii*, and two compounds in *L. schweinfurthii* var. *aschersonii*). Given the well-known, strong proximity of the three *Lycium* species, the criteria presented in this article should be useful in the identification and classification of these plants.

## Data availability statement

The original contributions presented in the study are included in the article/supplementary material. Further inquiries can be directed to the corresponding authors.

## Ethics statement

This article does not contain any studies with human participants or animals performed by any of the authors.

## Author contributions 

OR, RB, DM, IS, and ME: conceptualization. OR, RB, DM, SY, RO, and ME: methodology. OR, RB, AA, AE-S, and ME: software. OR, RB, DM, AA, and ME: validation. OR, RB, and ME: formal analysis. OR, RB, and ME: investigation. OR, RB, HE-R, AE-T, and ME: resources. OR, RB, SY, RO, ME, and AE-S: data curation. OR, RB, ME, and DM: writing—preparation of original draft. OR, RB, DM, IS, NG, AE-S, and ME: writing—review and editing. OR, RB, DM, HE-R, AE-T, and ME: visualization. OR, RB, IS, NG, AE-S, and ME: supervision. All authors contributed to the article and approved the submitted version.
